# Factors influencing patient disclosure of cancer diagnosis to the family dentist: online survey in Japan

**DOI:** 10.1038/s41598-022-22219-8

**Published:** 2022-10-17

**Authors:** Kyunghee Lee, Kozo Takase, Kiyohide Fushimi

**Affiliations:** 1 Department of Health Policy and Informatics, Graduate School of Medical and Dental Sciences, Tokyo Medical and Dental University, 1-5-45 Yushima, Bunkyo-Ku, Tokyo, 1138519 Japan; 2 Department of Research Development, Graduate School of Medical and Dental Sciences, Tokyo Medical and Dental University, Tokyo, Japan

**Keywords:** Breast cancer, Prostate cancer, Colorectal cancer, Gastric cancer, Lung cancer, Dentistry, Quality of life, Oncology

## Abstract

Oral care during cancer treatment constitutes essential supportive care. We aimed to identify factors associated with cancer patients informing their family dentists about their cancer diagnosis. Using the generated original questionnaire, we conducted a cross-sectional questionnaire study in 500 cancer patients (gastric, colorectal, lung, breast, and prostate cancer) through the Internet from September 10 to 13, 2019. The factors influencing patients’ disclosure of their cancer diagnosis to their family dentist were identified by multivariable logistic regression analysis. Nearly half of the respondents (42.2%) informed their family dentist that they had cancer. The disclosing behavior of cancer patients was distinctively associated with their physician’s advice (odds ratio [OR] 59.3; 95% confidence interval [CI] 7.7–456.3); 8.6% of all respondents were advised to inform their dentist about their cancer diagnosis. In the group without the physician's advice, good relationship with family dentist was associated with disclosing behavior. This study indicates the need for support for cancer patients to receive appropriate oral care; patients' perceptions of the physician’s advice and communication with the family dentist should be motivators for disclosing the cancer diagnosis to dentists. Medical workers involved in cancer care should demonstrate the benefit of medical-dental collaboration in cancer care of patients.

## Introduction

Various acute and chronic oral complications, including oral mucositis, changes in salivary volume and viscosity, dysgeusia or ageusia, and dental diseases or jaw osteonecrosis, occur during cancer treatment^1^. Oral complications have a significant functional and psychosocial impact, including pain, dysphagia, anxiety, and depression, on the quality of life of cancer patients^1–3^; they affect cancer treatment, particularly by altering its intensity or interrupting the treatment^3–5^. Several studies showed the benefits of starting professional dental care before cancer treatment^6–8^. Oral care during cancer treatment constitutes essential supportive care. Collaboration between the cancer treatment team and dental professionals is essential from the pre-treatment to post-treatment phase^9,10^, and awareness of family dentists about the patient’s impending cancer treatment can facilitate the predictive assessment and implementation of preventive measures against oral complications, including consultations for early interventions when oral complications occur, avoidance of undesirable dental treatment during cancer treatment, and planned dental treatment or referral to dentists specialized in oral care during cancer treatment. In April 2012, “perioperative oral care” was covered by public health care insurance in Japan, with the aim of promoting preoperative medical–dental collaboration for cancer patients to streamline perioperative treatment and prevent oral complications. Worldwide, between 2005 and 2015, the age-standardized incidence rates for all cancers combined increased in 174 of 195 countries or regions, whereas the age-standardized mortality rates decreased in 140 of 195 countries or regions^11^. Globally, cancer cases and survivors are increasing every year; the need for local dental clinics to provide supportive oral care to cancer patients in collaboration with cancer treatment hospitals is estimated to increase. However, dentists are often informed only after completion of the patient's cancer treatment. Thus, there may be factors that prevent cancer patients from disclosing their medical condition. For example, dental clinics in Japan have a special layout that differs from medical clinics; the former have no private rooms; instead, there are semi-private rooms or open spaces wherein patients’ personal information is conveyed. Therefore, cancer patients may find it stressful to talk about their disease in such an environment. However, there is insufficient information regarding the relationship between patients’ awareness and facilities of the dental clinic. The willingness to undergo cancer screening is associated with sociodemographic characteristics^12^; however, the relationship between disclosing cancer diagnosis to family dentists and patient characteristics has not been investigated and there is insufficient research on the cancer diagnosis disclosing behavior of patients in dental clinics. Except during the patient interview at the initial and return visits, dentists experience difficulty in collecting reliable information about a patient’s disease status unless it is voluntarily self-disclosed. To facilitate disclosure of cancer diagnosis for administering appropriate oral care during cancer treatment, it is crucial to clarify the behavioral motivations of cancer patients at dental clinics.

This study was conducted to identify factors influencing patients regarding disclosure of their cancer diagnosis to dentists.

## Results

### Demographics of the respondents and response tendency

In total, 2442 panelists between the ages of 27 and 80 years participated in this preparatory survey, and we received a total of 500 responses from 100 each cancer patients. Table 1 shows the demographics of the respondents. 191 (38.2%) participants informed their family dentist that they had cancer (Q4b), 97 (19.4%) indicated that they had cancer in the medical questionnaire at their family dental clinic (Q4c), and 211 (42.2%) answered "yes" to either of these questions (Q4b or Q4c). Furthermore, of the 43 (8.6%) respondents who had received their physician’s advice (Q4a), 42 (97.7%) respondents informed their family dentist that they had cancer (Q4b or Q4c).

**Table 1:** Demographic characteristics of all responders (n = 500).

	Total	Gastric cancer	Colorectal cancer	Lung cancer	Breast cancer	Prostate cancer
	(n = 500)	(n = 100)	(n = 100)	(n = 100)	(n = 100)	(n = 100)
Age (mean, SD)	62.9 ± 9.32	65.4 ± 8.06	61.7 ± 8.87	64.1 ± 7.53	53.8 ± 8.15	69.3 ± 5.79
**Gender, n (%)**						
Male	348 (69.6)	90 (90.0)	78 (78.0)	80 (80.0)	0 (0.0)	100 (100.0)
Female	152 (30.4)	10 (10.0)	22 (22.0)	20 (20.0)	100 (100.0)	0 (0.0)
Post-2013^†^, n (%)	423 (84.6)	86 (86.0)	87 (87.0)	93 (93.0)	80 (80.0)	77 (77.0)
**Stage, n (%)**						
0	47 (9.4)	10 (10.0)	5 (5.0)	6 (6.0)	20 (20.0)	6 (6.0)
I	196 (39.2)	48 (48.0)	17 (17.0)	55 (55.0)	44 (44.0)	32 (32.0)
II	107 (21.4)	19 (19.0)	26 (26.0)	13 (13.0)	23 (23.0)	26 (26.0)
III	71 (14.2)	9 (9.0)	40 (40.0)	10 (10.0)	7 (7.0)	5 (5.0)
IV	24 (4.8)	6 (6.0)	4 (4.0)	7 (7.0)	0 (0.0)	7 (7.0)
Don't know	55 (11)	8 (8.0)	8 (8.0)	9 (9.0)	6 (6.0)	24 (24.0)
**Treatment plan (multiple choice), n (%)**						
Surgery	444 (88.8)	96 (96.0)	99 (99.0)	83 (83.0)	100 (100.0)	66 (66.0)
Chemical treatment	156 (31.2)	11 (11.0)	37 (37.0)	27 (27.0)	49 (49.0)	32 (32.0)
Radiation therapy	125 (25.0)	1 (1.0)	5 (5.0)	10 (10.0)	57 (57.0)	52 (52.0)
Immunotherapy	25 (5.0)	0 (0.0)	1 (1.0)	6 (6.0)	6 (6.0)	12 (12.0)
Others	18 (3.6)	3 (3.0)	1 (1.0)	3 (3.0)	7 (7.0)	4 (4.0)
Familiar person, n (%)	285 (57.0)	55 (55.0)	65 (65.0)	58 (58.0)	52 (52.0)	55 (55.0)
**Treatment status, n (%)**						
Before treatment	6 (1.2)	1 (1.0)	0 (0.0)	1 (1.0)	0 (0.0)	4 (4.0)
Ongoing treatment	123 (24.6)	19 (19.0)	11 (11.0)	20 (20.0)	51 (51.0)	22 (22.0)
After treatment	367 (73.4)	79 (79.0)	87 (87.0)	78 (78.0)	49 (49.0)	74 (74.0)
Don't know	4 (0.8)	1 (1.0)	2 (2.0)	1 (1.0)	0 (0.0)	0 (0.0)
Dental clinic type is "single department clinic style", n (%)	463 (92.6)	93 (93.0)	95 (95.0)	93 (93.0)	90 (90.0)	92 (92.0)
**Family dentist, n (%)**						
Male	442 (88.4)	93 (93.0)	89 (89.0)	91 (91.0)	79 (79.0)	90 (90.0)
Female	38 (7.6)	5 (5.0)	8 (8.0)	6 (6.0)	13 (13.0)	6 (6.0)
Other	20 (4.0)	2 (2.0)	3 (3.0)	3 (3.0)	8 (8.0)	4 (4.0)
Private group, n (%)	313 (62.6)	57 (57.0)	67 (67.0)	62 (62.0)	72 (72.0)	55 (55.0)

^†^"Post-2013" is the responders who diagnosed cancer after 2013.

### Substructure and reliability of the questionnaire’s consciousness items

We conducted a factor analysis (principal factor method with varimax rotation) of the 23 items related to awareness in the questionnaire items (Table 2); six patient awareness factors were extracted as the substructure, whereas all 23 items were selected with an eigenvalue ≥ 1.

**Table 2:** Results of factor analysis for question items (n = 500).

Items		Factor					
		1	2	3	4	5	6
**(1) First factor: complications experience (α = 0.828, contribution ratio; 9.81%)**							
4g	Complications (pain, etc.)	0.821	0.020	0.077	0.044	− 0.085	− 0.127
4h	Complications (dry mouth)	0.841	0.051	0.100	0.023	− 0.147	− 0.099
4i	Complications (taste disorder)	0.657	0.139	0.004	0.049	− 0.001	− 0.025
**(2) Second factor: health knowledge (α = 0.707, contribution rate; 8.90%)**							
2a	Knowledge of healthy life expectancy	− 0.252	0.282	0.126	0.223	− 0.042	− 0.100
2b	Knowledge of 8020 promotion	− 0.239	0.358	0.078	0.105	− 0.100	− 0.034
2c	Knowledge of Medical and Dental Care Cooperation	0.134	0.629	0.114	0.115	− 0.028	0.094
2d	Keeping oral hygiene	0.073	0.780	0.080	0.111	0.205	0.138
2e	Causes oral complications	0.180	0.777	− 0.001	0.122	0.231	0.019
**(3) Third factor: positive attitude toward maintaining health (α = 0.671, contribution rate; 8.84%)**							
5a	Regular check-up	− 0.205	0.141	0.446	0.161	0.107	0.078
5c	Relationship between treatments	0.150	0.037	0.656	0.320	0.147	− 0.118
5d	Dentist opinion	0.201	0.044	0.717	0.293	0.158	0.021
5 g	Conscious of life	0.074	0.127	0.321	0.117	0.047	− 0.216
**(4) Fourth factor: positive opinion of cancer medical-dental collaboration (α = 0.767, contribution rate; 7.58%)**							
3a	Cooperate between doctors	0.025	0.274	0.256	0.725	0.214	− 0.050
3b	Automatically health data system	0.020	0.134	0.260	0.726	0.159	0.037
3c	Tell the dentist	0.031	0.127	0.198	0.485	0.163	0.091
**(5) Fifth factor: opinion about the relationship between body and mouth (α = 0.739, contribution rate; 5.90%)**							
3d	Need to know	− 0.072	0.001	0.171	0.195	0.608	0.096
3e	Direct relationship	− 0.046	0.155	0.199	0.209	0.747	− 0.003
**(6) Sixth factor: good relationship with family dental clinic (α = 0.589, contribution rate; 5.80%)**							
4e	Other patients	− 0.140	− 0.098	− 0.248	− 0.101	0.061	0.518
4f	Privacy	0.102	0.073	0.037	0.026	− 0.048	0.479
4j	Interest of dentist	− 0.188	0.083	0.043	0.054	0.267	0.288
5b	Satisfaction	− 0.251	0.051	0.438	− 0.022	0.043	0.500
5e	Dentist care	0.008	0.054	0.474	0.132	0.088	0.485
5f	Asks from dentist	− 0.141	0.101	− 0.082	0.083	0.217	0.336

All Items; α = 0.759.

Cumulative contribution ratio; 46.83%.

### Factors associated with informing family dentist about cancer diagnosis

Univariable analysis in all respondents that included 35 items showed a significant difference regarding 25 items (*p* < 0.1; Supplementary Table 1). To avoid overfitting, we selected 20 of the 25 items selected by univariable analysis as relevant to this study's purpose. These selected items indicated correlation coefficients within the range of − 0.25 to + 0.77. The above-mentioned 20 items were included as independent variables in the multiple logistic regression analysis (*p* < 0.05; Table 3), and significant differences were found in four items. “Physician advice” (Q4a) had an odds ratio (OR) of 59.3 (95% confidence interval [CI] 7.7–456.3, *p* < 0.001) and “asks from dentist”(Q5f) had an OR of 2.04 (95% CI 1.50–2.77, *p* < 0.001).

**Table 3:** Results of multivariable logistic regression analysis in all respondents (n = 500).

Items		Partial regression coefficient	OR (95% CI)	p
Gender (male)		− 0.43	0.65 (0.40–1.07)	0.09
Post-2013		0.41	1.51 (0.80–2.82)	0.20
2d	Keeping oral hygiene	0.11	1.12 (0.82–1.52)	0.49
2e	Causes oral complications	0.09	1.09 (0.80–1.48)	0.58
3a	Cooperate between doctors	− 0.19	0.83 (0.52–1.32)	0.43
3c	Tell the dentist	0.67	1.95 (1.32–2.87)	P < 0.001**
3e	Direct relationship	0.04	1.04 (0.74–1.46)	0.82
4a	Physician advice	4.08	59.33 (7.71–456.34)	P < 0.001**
4e	Other patients	0.31	1.37 (0.98–1.92)	0.07
4f	Privacy	− 0.14	0.87 (0.63–1.19)	0.38
4g	Complications (pain, etc.)	0.30	1.35 (0.82–2.23)	0.24
4h	Complications (dry mouth)	0.19	1.21 (0.70–2.12)	0.50
4i	Complications (taste disorder)	0.26	1.30 (0.95–1.78)	0.10
5a	Regular check-up	0.32	1.38 (0.87–2.20)	0.17
5b	Satisfaction	0.16	1.18 (0.73–1.90)	0.51
5c	Relationship between treatments	− 0.33	0.72 (0.45–1.16)	0.18
5d	Dentist opinion	0.15	1.16 (0.71–1.91)	0.55
5e	Dentist care	0.53	1.69 (1.13–2.54)	0.011*
5f	Asks from dentist	0.71	2.04 (1.50–2.77)	P < 0.001**
5g	Conscious of life	0.23	1.26 (0.94–1.69)	0.12

*P < 0.05, **P < 0.01.

Nagelkerke R^2^; 0.39.

The percentage of correct classifications; 75.0%.

### Factors associated with informing family dentist about cancer diagnosis in the group without physician's advice

A chi-square test comparing the groups with (n = 43) and without physician's advice (n = 457) showed a higher frequency of informing the dentist about cancer diagnosis (42/43) in the former than the latter (*p* < 0.001).

To identify factors related to the disclosing behavior of respondents who did not receive advice from their physicians, we conducted a univariable analysis focusing on this group (*p* < 0.1; Supplementary Table 1). This univariable analysis used 34 items except for Q4a and found a significant difference (*p* < 0.1) regarding 21 items. The private group (*p* = 0.6), other patients around the treatment seat (including "agree slightly"; *p* = 0.15; Q4e), and no privacy at the treatment seat (including "agree slightly;" *p* = 0.15; Q4f) did not differ between the disclosing and non-disclosing groups. We extracted 16 of the 21 items selected in the univariable analysis that were significant for the same variables used in all respondents' multiple logistic regression analysis. These 16 items indicated correlation coefficients within the range of − 0.27 to + 0.76. The above-mentioned 16 items were included as independent variables in the multiple logistic regression analysis (*p* < 0.05; Table 4), and significant differences were found in four items: “asks from dentist” (Q5f) had an OR of 2.10 (95% CI 1.56–2.84, *p* < 0.001), “tell the dentist” (Q3c) had an OR of 1.79 (95% CI 1.22–2.62, *p* = 0.0027), and “dentist care” (Q5e) had an OR of 1.76 (95% CI 1.18–2.64, *p* = 0.0061). "Post-2013" indicated the difference in univariable analysis (p = 0.065) but not in multiple logistic regression analysis (OR 1.47; 95% CI 0.79–2.74; p = 0.22).

**Table 4:** Results of multivariable logistic regression analysis in the group without physician's advice (n = 457).

Items		Partial regression coefficient	OR (95% CI)	p
Gender (Male)		− 0.33	0.72 (0.44–1.16)	0.17
Post-2013		0.39	1.47 (0.79–2.74)	0.22
2d	Keeping oral hygiene	0.15	1.16 (0.85–1.57)	0.35
2e	Causes oral complications	0.04	1.04 (0.77–1.41)	0.79
3a	Cooperate between doctors	− 0.19	0.83 (0.52–1.31)	0.42
3c	Tell the dentist	0.58	1.79 (1.22–2.62)	0.0027**
3e	Direct relationship	0.08	1.09 (0.78–1.52)	0.63
4g	Complications (pain, etc.)	0.37	1.44 (0.87–2.39)	0.15
4h	Complications (dry mouth)	0.04	1.04 (0.60–1.81)	0.89
4i	Complications (taste disorder)	0.33	1.39 (1.01–1.90)	0.041*
5a	Regular check-up	0.38	1.47 (0.92–2.33)	0.11
5b	Satisfaction	0.10	1.10 (0.69–1.75)	0.69
5c	Relationship between treatments	− 0.29	0.75 (0.47–1.21)	0.24
5d	Dentist opinion	0.15	1.16 (0.71–1.90)	0.56
5e	Dentist care	0.57	1.76 (1.18–2.64)	0.0061**
5f	Asks from dentist	0.74	2.10 (1.56–2.84)	P < 0.001**

*P < 0.05, **P < 0.01.

Nagelkerke R^2^; 0.27.

The percentage of correct classifications; 73.7%.

Further, we conducted a multivariable logistic regression analysis using the six patient-awareness factors to focus on the group without physician's advice (*p* < 0.05; Table 5). The correlation coefficient between each factor was |r| ≤ 0.164. There were significant associations (*p* < 0.05) for five factors. Factor 6 (good relationship with the family dentist) had an OR of 1.88 (95% CI 1.43–2.48; *p* < 0.001), and factor 4 (positive opinion of cancer medical-dental collaboration) had an OR of 1.25 (95% CI 0.97–1.62; *p* = 0.081).

**Table 5:** Results of multivariable logistic regression analysis in the group without physician’s advice (n = 457).

Items		Partial regression coefficient	OR (95% CI)	p
Factor 1	Complications experience	0.38	1.46 (1.16–1.83)	0.001**
Factor 2	Health knowledge	0.35	1.42 (1.12–1.78)	0.003**
Factor 3	Positive attitude toward maintaining health	0.28	1.32 (1.03–1.70)	0.029*
Factor 4	Positive opinion of cancer medical-dental collaboration	0.23	1.25 (0.97–1.62)	0.081
Factor 5	Opinion about the relationship between body and mouth	0.36	1.44 (1.11–1.87)	0.007**
Factor 6	Good relationship with family dental clinic	0.63	1.88 (1.43–2.48)	P < 0.001**

*P < 0.05, **P < 0.01.

Nagelkerke R^2^; 0.17.

The percentage of correct classifications; 68.7%.

## Discussion

We identified factors that influence cancer patients' disclosing behavior in dental clinics. The main finding was that patients' perception of being recommended by their physicians was distinctively related to it, and a good relationship with the family dentist was associated too.

The six patient awareness factors identified by the factor analysis were as expected in the substructure of the questionnaire items. The reliability test showed that despite the relatively low Cronbach's α for factor 6, the Cronbach's α for other factors was generally high and adequate for all items.

The results of multivariable logistic regression analysis for all respondents showed that physician’s advice (Q4a) was distinctively associated with the disclosing behavior of cancer patients at dental clinics. The logistic regression analysis showed that inquiries from dentist (Q5f) were associated with the disclosing behavior, although physician’s advice was distinctively associated with informing the dentist. This result supports the findings of previous studies^13,14^ that physician’s recommendations motivate patients and are associated with willingness to undergo cancer screening. We revealed that the patient's perception of being recommended by their physicians was a strong motivator for patients’ disclosing behavior. Cancer patients are less aware of their oral health due to fear of death and anxiety about treatment^15^. Oral care during cancer treatment contributes to improving the QOL of cancer patients and reducing healthcare costs^9^ by reducing oral complications^6–8^, postoperative pneumonia^16–18^, and duration of hospital stay^19,20^. It is necessary to fully inform patients of the significance of oral care and the benefits of medical–dental collaboration, and to link this to patient behavior. Only 8.6% of our respondents were advised by their physician; this indicates the need to encourage attending physicians to advise their patients.

The test results for the attending physician’s advice and the frequency of disclosing showed that the group with physician's advice differed from the group without physician's advice; thus, we evaluated the group without physician's advice. Univariable analysis showed that the facilities of the dental clinic facilities do not affect disclosing behavior. Public health care insurance coverage of "perioperative oral care" had little impact on disclosing behavior. It may be necessary to inform more cancer patients and medical workers that patients can receive "perioperative oral care" for a small financial contribution. The results of two multiple logistic regression analyses conducted on this group showed that patients' perception of good communication with their family dentist was significantly associated with disclosing behavior. The OR of "good relationship with the family dentist" was higher than that of other factors. It suggests that establishing good communication may significantly influence disclosing behavior more than other factors. "Complications experience", "health knowledge", "Positive attitude toward maintaining health", and "opinion about the relationship between body and mouth" reflect personal experiences, knowledge, opinions, and characteristics of patients, and it is difficult for family dentists to be directly involved in these. Therefore, dentists should regularly establish good communication with their patients. Previous studies^21,22^ reported that the physician’s communication style can alleviate patients' anxiety and improve satisfaction. We revealed that patients' perceptions of good communication with their dentists help them receive appropriate oral care.

## Limitations

This study’s cross-sectional design precludes the detection of a causal relationship, and the change in awareness of health and oral care that occurred after disclosing cancer diagnosis to the family dentist may have influenced the responses. The results of this study do not facilitate a discussion of exclusion criteria or examination of factors other than those specified in the questionnaire. This study does not reflect differences in the distribution of cancers.

## Conclusion

This cross-sectional questionnaire study was conducted through the Internet. The main finding was that patients' perception of being recommended by their physicians was distinctively related to cancer patients' disclosing behavior in dental clinics. We revealed that communication from medical workers was a motivating factor for disclosing behavior of patients. Medical workers involved in cancer care are required to demonstrate the benefit of medical-dental collaboration to patients.

## Methods

### Study population and study design

Online surveys facilitate access to many potential respondents, especially those from hidden populations^23^. The internet population-penetration rate in Japan was 83.5% in 2016^24^; thus, we decided to use an online survey. We conducted an online questionnaire survey from September 10 to 13, 2019 through an online-research company (Rakuten Insight, Inc.^25^) among panelists registered with this company. This company (Rakuten Insight, Inc.^25^) recruits and has various disease panelists for its own and other companies' online surveys. The panelists participate freely in various surveys sent by this company, and they get points as rewards for answering the survey questions. As of January 2019 (survey period November 21–December 20, 2018), there were 18,543 cancer patients among disease panelists, of which 9349 (50.42%) had five common cancers with high incidence as reported by the Cancer Information Service of the National Cancer Center Japan^26^. In this study, patients with the five most common cancers with high incidence, as reported by the Cancer Information Service of the National Cancer Center Japan^26^, were included in the registry to ensure the number of responses and facilitate interpretation of the results. We included only those respondents whose family dental clinic was the same as that before they were diagnosed with cancer. This is because our study focuses on whether patients disclose their cancer diagnosis during their return visit to the dental clinic. The exclusion criteria were: cancer recurrence, cancer types not covered by this study, multiple cancers, and "current family dental clinic is not the clinic the respondents visited before the onset of cancer."

First, we conducted a preparatory survey (Supplementary Table 2) through an online-research company (Rakuten Insight, Inc.^25^). Second, we conducted our main survey (Supplementary Table 2) through this company. Before the preparatory survey, it was stated that our study might contain personal information items that require consideration, the responses would be used only for analysis in this study, the responses would not be used to identify the respondents or for advertising or sales promotion, and the respondents should only participate in this study if they agreed to its conditions. In both the preparatory and main surveys, all questions were mandatory, and the respondents were allowed to leave the study at any time while answering the questions. The questionnaires in which all items were answered were counted as valid questionnaires. We terminated the main survey for each cancer-type when the number of respondents reached 100.

The preparatory and main surveys were conducted by an online-research company (Rakuten Insight, Inc.^25^) that provided only anonymized data. We did not communicate directly with the respondents and any personal identifiable information was not obtained. This study was approved by the ethics committee of Tokyo Medical and Dental University in February 2019 (M2018–2236).

### Questionnaire development

We generated the original questionnaire used in this study based on previous reports^21,27^ and clinical experience of patients. The questionnaire of the main survey comprised 34 items from the five sections (Supplementary Table 2).

### Variable processing

The dependent variable in this study was the disclosure or no disclosure; therefore, respondents who answered “yes” to either of the following two questions and those who answered “no” to both questions were assigned to the disclosing group and the non-disclosing group, respectively: Q4b: “Did you tell your family dentist that you had cancer?” and Q4c: “Did you mention in the medical questionnaire at your family dental clinic that you had cancer?” We included Q4c because in Japan, patients are often asked to fill medical questionnaires at return visits after a while.

To investigate the effect of public health care insurance coverage of "perioperative oral care," we created "Post-2013" as variables based on Q1a (Timing of diagnosis). "Post-2013" is the responders diagnosed with cancer after 2013.

To investigate the effect of the dental clinic facilities, we created private group and non-private group as dummy variables based on whether the patient's line of sight was blocked using question Q4d: “Is there anything between the treatment seats at your family dental clinic?” Patients who answered “private room, wall, curtain, or partition” and “no machine or no partition” were assigned to the private group and non-private group, respectively. Moreover, as the answer to this question included “others (free comments),” the patient answering “reservation for only one person” and “some private rooms” was assigned to the private group and non-private group, respectively.

Reverse item processing was performed to facilitate the interpretation of results of questions Q3d, Q3e, Q4e, Q4j, and Q5f. For the response items using sores 4 to 1, the analysis was conducted with scores 4 or 3 considered as “know/agree” and scores 2 or 1 as “do not know/disagree.”

### Statistical analysis

All statistical analyses were conducted using the BellCurve for Excel version 3.20 (Social Survey Research Information Co., Ltd, Tokyo, Japan). We conducted a factor analysis on 23 items related to awareness to confirm the questionnaire’s supposed substructure (principal factor method with varimax rotation). We calculated Cronbach’s alpha to investigate reliability of the extracted factors (six factors as patient awareness factors). We performed a univariable analysis of 35 variables, including the above-mentioned 23 items plus gender, age, and environment of the dental treatment seat, to identify items related to the dependent variable in this study (*p* < 0.1). The multivariable logistic regression analysis included variables considered significant as per univariable logistic analysis (*p* < 0.05).

Respondents who answered yes or no to Q4a were assigned to the group with physician’s advice and the group without physician’s advice, respectively, and a chi-square test was used to compare both groups for disclosure or no disclosure (*p* < 0.05). Next, we conducted a univariable analysis using the above-mentioned 34 items except for Q4a against the group without physician’s advice. The multivariable logistic regression analysis included variables identified as significant on univariable logistic analysis. Further, we conducted multivariable logistic regression analysis for the relationship between factor scores introduced by the factor analysis and disclosure in the group without the physician’s advice.

### Research involving human participants

This study was approved by the appropriate institutional research ethics committee of Tokyo Medical and Dental University in February 2019 (M2018-236). The authors certify that the study was performed in accordance with the ethical standards as laid down in the 1964 Declaration of Helsinki and its later amendments or comparable ethical standards. An informed consent was obtained from all subjects/participants.

### Consent to participate

All responders participated by an opt-in method through an online research company.

## Supplementary Information

Supplementary Tables.

## Data Availability

The data used to support the findings of this study are available from the corresponding author upon reasonable request.
