# Supplementary material for: Factors influencing patient disclosure of cancer diagnosis to the family dentist: online survey in Japan
Source: Sci Rep. 2022 Oct 17;12:17375. doi: 10.1038/s41598-022-22219-8 (PMC9576789; doi:10.1038/s41598-022-22219-8)
Supplement: Supplementary file 1 — Supplementary Tables. [file 41598_2022_22219_MOESM1_ESM.docx]

**Factors influencing patient disclosure of cancer diagnosis to the family dentist: online survey in Japan.**

Supplementary Material

**Supplementary Table 1** Results of univariable analysis

| Items (n, %) | | All respondents  (n=500) | |  |  | The group without physician's advice (n=457) | | | Factor analysis result |
| --- | --- | --- | --- | --- | --- | --- | --- | --- | --- |
|  |  |  |  |  |  |  |  |  |  |
|  |  | Disclosing group | Non-disclosing group | p |  | Disclosing group | Non-disclosing group | p |  |
|  |  | n=211  (42.2) | n=289  (57.8) |  |  | n=169  (37.0) | n=288  (63.0) |  |  |
| Gender | |  |  |  |  |  |  |  |  |
| Male | | 138(65.4) | 210(72.7) | **0.082^*^** |  | 109(64.5) | 209(72.6) | **0.07*** | ― |
| Female | | 73(34.6) | 79(27.3) |  |  | 60(35.5) | 79(27.4) |  |  |
| Age (mean, SD) | | 62.3±9.2 | 62.3±9.4 | 0.24 |  | 62.4±9.3 | 63.2±9.4 | 0.35 | ― |
| Post-2013^†^ | | 189(89.6) | 234(81) | **0.0084^*^** |  | 148(87.6) | 233(80.9) | **0.065*** | ― |
| Treatment plan (multiple choice）(Q1c) | |  |  |  |  |  |  |  |  |
| Surgery | | 190(90) | 254(87.9) | 0.45 |  | 150(88.8) | 253(87.8) | 0.77 | ― |
| Chemical treatment | | 80(37.9) | 76(26.3) | **0.0056^*^** |  | 63(37.3) | 75(26.0) | **0.011*** | ― |
| Radiation therapy | | 57(27) | 68(23.5) | 0.38 |  | 46(27.2) | 68(23.6) | 0.39 | ― |
| Immunotherapy | | 12(5.7) | 13(4.5) | 0.55 |  | 8(4.7) | 13(4.5) | 0.91 | ― |
| Others | | 6(2.8) | 12(4.2) | 0.44 |  | 5(3.0) | 12(4.2) | 0.51 | ― |
| Familiar person (Q1d) | | 124(58.8) | 161(55.7) | 0.50 |  | 100(59.2) | 161(55.9) | 0.50 | ― |
| Dental clinic type is "Single department clinic" (Q1f) | | 195(92.4) | 268(92.7) | 0.89 |  | 158(93.5) | 267(92.7) | 0.75 | ― |
| Private group | | 135(64.0) | 178(61.6) | 0.51 |  | 108(63.9) | 177(61.5) | 0.6 | ― |
| 4a | Physician advice | 42(19.9) | 1(0.3) | **<0.001^*^** |  | ― | ― | ― | ― |
| 4g | Complications (pain, etc.) | 41(19.4) | 21(7.3) | **<0.001^*^** |  | 29(17.2) | 20(6.9) | **0.009*** | Factor 1 |
| 4h | Complications (dry mouth) | 30(14.2) | 17(5.9) | **0.007^*^** |  | 19(11.2) | 17(5.9) | **0.059*** | Factor 1 |
| 4i | Complications (taste disorder) | 54(25.6) | 30(10.4) | **<0.001^*^** |  | 40(23.7) | 29(10.1) | **<0.001*** | Factor 1 |
| 2a | Knowledge of healthy life expectancy | 171(81) | 243(84.1) | 0.96 |  | 139(82.2) | 242(84.0) | 0.71 | Factor 2 |
| 2b | Knowledge of 8020 promotion | 138(65.4) | 168(58.1) | 0.18 |  | 113(66.9) | 167(58.0) | 0.12 | Factor 2 |
| 2c | Knowledge of Medical and Dental Care Cooperation | 49(23.2) | 24(8.3) | **<0.001^*^** |  | 31(18.3) | 23(8.0) | **0.024*** | Factor 2 |
| 2d | Keeping oral hygiene | 123(58.3) | 101(34.9) | **<0.001^*^** |  | 91(53.8) | 100(34.7) | **<0.001*** | Factor 2 |
| 2e | Causes oral complications | 115(54.5) | 96(33.2) | **<0.001^*^** |  | 87(51.5) | 95(33.0) | **<0.001*** | Factor 2 |
| 5a | Regular check-up | 207(98.1) | 269(93.1) | **<0.001^*^** |  | 166(98.2) | 268(93.1) | **<0.001*** | Factor 3 |
| 5c | Relationship between treatments | 150(71.1) | 173(59.9) | **0.033^*^** |  | 122(72.2) | 172(59.7) | **0.040*** | Factor 3 |
| 5d | Dentist opinion | 140(66.4) | 134(46.4) | **<0.001^*^** |  | 112(66.3) | 133(46.2) | **<0.001*** | Factor 3 |
| 5g | Conscious of life | 160(75.8) | 201(69.6) | **0.031^*^** |  | 127(75.1) | 200(69.4) | 0.16 | Factor 3 |
| 3a | Cooperate between doctors | 189(89.6) | 218(75.4) | **<0.001^*^** |  | 150(88.8) | 217(75.3) | **<0.001*** | Factor 4 |
| 3b | Automatically health data system | 162(76.8) | 194(67.1) | **0.027^*^** |  | 131(77.5) | 193(67.0) | **0.061*** | Factor 4 |
| 3c | Tell the dentist | 175(82.9) | 167(57.8) | **<0.001^*^** |  | 140(82.8) | 166(57.6) | **<0.001*** | Factor 4 |
| 3d | Need to know | 155(73.5) | 188(65.1) | **0.002^*^** |  | 126(74.6) | 187(64.9) | **0.0012*** | Factor 5 |
| 3e | Direct relationship | 168(79.6) | 195(67.5) | **<0.001^*^** |  | 136(80.5) | 194(67.4) | **<0.001*** | Factor 5 |
| 4e | Other patients | 155(73.5) | 203(70.2) | **0.095^*^** |  | 124(73.4) | 203(70.5) | 0.15 | Factor 6 |
| 4f | Privacy | 108(51.2) | 117(40.5) | **0.014^*^** |  | 78(46.2) | 117(40.6) | 0.15 | Factor 6 |
| 4j | Interest of dentist | 142(67.3) | 165(57.1) | **0.012^*^** |  | 111(65.7) | 164(56.9) | **0.055*** | Factor 6 |
| 5b | Satisfaction | 186(88.2) | 242(83.7) | **0.0028^*^** |  | 150(88.8) | 242(84.0) | **0.0038*** | Factor 6 |
| 5e | Dentist care | 160(75.8) | 158(54.7) | **<0.001^*^** |  | 129(76.3) | 157(54.5) | **<0.001*** | Factor 6 |
| 5f | Asks from dentist | 174(82.5) | 188(65.1) | **<0.001^*^** |  | 142(84.0) | 187(64.9) | **<0.001*** | Factor 6 |
| *：P<0.1 | | | | | | | | | |
| † "Post-2013" is the responders who diagnosed cancer after 2013. (Q1a) | | | | | | | | | |

**Supplementary Table 2** Question list of preparatory survey and main survey

| No. | | Labels | Questions | Answer categories |
| --- | --- | --- | --- | --- |
| 1. Preparatory survey | | |  |  |
|  | SC1 | Cancer type | What types of cancers have you had in your life? | (multiple choice） ①gastric cancer, ②colorectal cancer, ③lung cancer, ④breast cancer, ⑤prostate cancer, ⑥other cancer, ⑦I have never had cancer |
|  |  |  |  | The conditions for exit are multiple responses or answer ⑥ or ⑦ |
|  | SC2 | Cancer recurrence | Have you ever had a recurrence of the cancer you answered in the previous question? | ①yes, ②no, ③don't know |
|  |  |  |  | The conditions for exit are answer ① or ③ |
|  | SC3 | Family dental clinic(1) | Do you have a family dental clinic? | ①yes, ②no, ③don't know |
|  |  |  |  | The conditions for exit are answer ② or ③ |
|  | SC4 | Family dental clinic(2) | Is the family dental clinic you answered in the previous question the one you have been going to since before you had cancer? | ①yes, ②no, ③don't know |
|  |  |  |  | The conditions for exit are answer ② or ③ |
| 2. Main survey | | |  |  |
| 1) Attribute section; attribute of responders such as the year of cancer diagnosis and cancer treatment. | | | | |
|  | 1a | Timing of diagnosis | When was the first time you were diagnosed you had cancer? | year and month |
|  | 1b | Stage | What was the stage of your cancer when you were first diagnosed with it? | 0, I, II, III, IV, don't know |
|  | 1c | Treatment plan | What was included in your cancer treatment plan when you were first diagnosed with cancer? | (multiple choice）surgery, chemical treatment, radiation therapy, immunotherapy, other(free comments) |
|  | 1d | Familiar person | When you were first diagnosed with cancer, was there anyone close to you who had experienced cancer? | yes, no |
|  | 1e | Treatment status | What is the current status of your cancer treatment? | before treatment, among treatment, after treatment, don't know |
|  | 1f | Dental clinic type | Which type is your family dental clinic? | single dental clinic, dental clinic with other departments, dental department in a large hospital such as a general hospital, other(free comments) |
|  | 1g | Family dentist | What is the gender of your family dentist? | male, female, other^‡^ |
| 2) Knowledge section; knowledge about general and oral health. | | | | |
|  | 2a | Knowledge of healthy life expectancy | You know the meaning of "healthy life expectancy". | four choices^†^ |
|  | 2b | Knowledge of 8020 promotion | You know the meaning of "8020 promotion^*^". | four choices^†^ |
|  | 2c | Knowledge of medical-dental collaboration | You know the meaning of medical–dental collaboration for cancer care. | four choices^†^ |
|  | 2d | Keeping oral hygiene | You know that it is important to maintain oral hygiene before and after cancer surgery. | four choices^†^ |
|  | 2e | Causes oral complications | You know that cancer treatments can cause oral complications (stomatitis, taste disorders, etc.) | four choices^†^ |
| 3) Opinion section; opinion about handling health information of cancer patients by medical/dental/healthcare professionals. | | | | |
|  | 3a | Cooperate between doctors | You agree that your cancer physician and your family dentist cooperates to treat your cancer. | four choices^†^ |
|  | 3b | Automatically health data system | You agree on a system in which your health information (cancer, etc.) is automatically sent to your family dental clinic. | four choices^†^ |
|  | 3c | Tell the dentist | You want to tell your health information (history of cancer, etc.) to your family dental clinic yourself | four choices^†^ |
|  | 3d | Need to know | You don't think that your family dental clinic does not need to know your health information (history of cancer, etc.). | four choices^†^ |
|  | 3e | Direct relationship | You don't think there isn't a direct relationship between the teeth and mouth and body. | four choices^†^ |
| 4) Experience section; experience after cancer diagnosis. | | | | |
|  | 4a | Physician advice | Were you advised by your cancer physician to tell your family dental clinic that you had cancer? | yes, no |
|  | 4b | Talked the dentist | Did you tell your family dentist that you had cancer? | yes, no |
|  | 4c | Wrote in questionnaire | Did you mention in a medical questionnaire at your family dental clinic that you had cancer? | yes, no |
|  | 4d | Around the sheet | Is there anything between the treatment sheets in your family dental clinic? | private room, wall, curtain, partition, no machine, no partition, other(free comments) |
|  | 4e | Other patients | You aren't concerned about other patients when you talk to your family dentist on the dental chair. | four choices^†^ |
|  | 4f | Privacy | You feel that your privacy is secured when on the dental chair at your family dental clinic. | four choices^†^ |
|  | 4g | Complications (pain, etc.) | You felt troubled with cancer complications such as pain, bleeding, inflammation, of the teeth, gums, tongue, etc. | four choices^†^ |
|  | 4h | Complications (dry mouth) | You felt troubled with dry mouth as a complication of cancer. | four choices^†^ |
|  | 4i | Complications (taste disorder) | You felt troubled with taste disorder as a complication of cancer. | four choices^†^ |
|  | 4j | Interest of dentist | You don't think that your family dentist seems not interested in your health information (cancer, etc.). | four choices^†^ |
| 5) Characteristic section; thoughts on health and oral health. | | | | |
|  | 5a | Regular check-up | You think it is necessary to have a regular check-up at your family dental clinic. | four choices^†^ |
|  | 5b | Satisfaction | You are satisfied with your dentist as your family dentist. | four choices^†^ |
|  | 5c | Relationship between treatments | You want to know information about the relationship between cancer treatment and dental treatment. | four choices^†^ |
|  | 5d | Dentist opinion | You want to know the opinions of your family dentist regarding the maintenance of dental and oral health of cancer patients. | four choices^†^ |
|  | 5e | Dentist care | You feel that your family dentist cares about you well. | four choices^†^ |
|  | 5f | Asks from dentist | You will tell to your family dentist that you had cancer, if your dentist asks you "Did something change in your body?". | four choices^†^ |
|  | 5g | Conscious of life | By had cancer, you became more conscious of your life. | four choices^†^ |
| † The choices are "4(strongly know/strongly agree)-3(know a little/agree a little)-2(do not know a little/disagree a little)-1(strongly do not know/strongly disagree)". | | | | |
| ‡ We made this response option because sometimes we have multiple dentists at the dental clinic working on a single patient in japan. | | | | |
| * “8020 promotion" is set out to help people to keep 20 or more of their own teeth until the age of 80, in Japan. | | | | |
